# Splenic structural and functional abnormalities in individuals with *NR5A1/SF-1* variants

**DOI:** 10.1530/EC-25-0549

**Published:** 2025-12-16

**Authors:** Khadidja Fouatih, Camille Roussel, Maryse Cartigny, Muriel Houang, Lise Duranteau, Zeina Chakhtoura, Anne-Sophie Lambert, Marie-Agathe Trouvin, Barbara Girerd, Ekaterina Belozertseva, Delphine Borgel, Stephanie Franchi-Abella, Jérôme Bouligand, Maureen Lopez, Kenneth Chappell, Pierre Buffet, Ines Belguith, Claire Bouvattier, Abd El Kader Ait Tayeb

**Affiliations:** ^1^ Service d’endocrinologie et du diabète de l’enfant, Hôpital Bicêtre, Paris, France; ^2^ Centre de Référence des Maladies Rares du Développement Génital (CRMR DEVGEN) de l’hôpital Bicêtre, APHP Université Paris-Saclay, Le Kremlin-Bicetre, France; ^3^ Laboratoire d'Hématologie Générale, Hôpital Necker, APHP & UMR_S1134 – Inserm – Université Paris Cité, Paris, France; ^4^ Service d’Endocrinologie Pédiatrique, CHU Lille, Lille, France; ^5^ Explorations Fonctionnelles Endocriniennes, Centre de Référence Maladies Endocriniennes Rares de la Croissance et du Développement – CRESCENDO, Hôpital Armand Trousseau, APHP, Paris, France; ^6^ Service de Gynécologie médicale, Centre de Référence des Maladies Rares du Développement Génital (CRMR DEVGEN), Hôpital Bicêtre, APHP, Le Kremlin-Bicetre, France; ^7^ Service d’Endocrinologie et Médecine de la Reproduction, Hôpital La Pitié-Salpêtrière, Paris, France; ^8^ INSERM UMR-S U1185, Faculté de Médecine, University Paris-Saclay, Le Kremlin Bicêtre, France; ^9^ Service d’imagerie médicale pédiatrique, Hôpital Bicêtre, Le Kremlin-Bicetre, France; ^10^ APHP Université Paris-Saclay, Le Kremlin-Bicetre, France; ^11^ Service de Génétique Moléculaire, Pharmacogénétique et Hormonologie de Bicêtre, Hôpitaux Universitaires Paris-Saclay, Assistance Publique-Hôpitaux de Paris, Hôpital de Bicêtre, Le Kremlin Bicêtre, France; ^12^ Service des Maladies Infectieuses et Tropicales, Hôpital Necker, APHP & UMR_S1134 – Inserm – Université Paris Cité, Paris, France; ^13^ Centre de Recherche Clinique, Hôpitaux Universitaires Paris-Saclay, Assistance Publique-Hôpitaux de Paris, Hôpital de Bicêtre, Le Kremlin Bicêtre, France

**Keywords:** SF-1/NR5A1, disorders of sex development (DSD), spleen development, hyposplenism, asplenism

## Abstract

**Purpose:**

Steroidogenic factor 1 (SF-1), encoded by *NR5A1*, is essential for spleen development and function. *NR5A1* variants have been linked to abnormal spleen development. Hyposplenism exposes individuals to severe complications with potentially serious sequelae. This study aimed to determine the prevalence and principal features of hyposplenism in a cohort of French patients with *NR5A1* variants.

**Methods:**

We conducted a cross-sectional multicentre ancillary study among 34 patients carrying heterozygous *NR5A1* variants within the GR-EX cohort, which includes individuals from families affected by red blood cell diseases. All participants underwent splenic imaging (ultrasound, CT, or MRI) and pocked red blood cell (pRBC) quantification. pRBC thresholds of <7%, 7–20%, and >20% corresponded to normal splenic function, moderate hyposplenism, and severe hyposplenism, respectively. The primary endpoints were the prevalence of hyposplenism and its severity.

**Results:**

Functional hyposplenism was observed in 21/34 patients (61.7%), including 16/34 (47%) with severe forms. Morphological spleen abnormalities were identified in 15/34 patients (44.1%), with asplenia in 4/34 (11.7%). All patients with morphological spleen abnormalities on imaging also presented functional hyposplenism. Conversely, 6/21 patients (28%) with functional hyposplenism showed no morphological abnormalities on imaging. No association was found between *NR5A1* genotypes, gonadal phenotypes, and splenic anomalies.

**Conclusions:**

Functional hyposplenism was frequent in this cohort of patients carrying *NR5A1* variants, regardless of genotype and gonadal phenotype. Assessing splenic function is mandatory to help manage these patients. Preventive measures are also critical when hyposplenism is present.

## Introduction

Steroidogenic factor 1 (SF-1), also known as nuclear receptor subfamily 5 group A member 1 (NR5A1), is a nuclear receptor encoded by the *NR5A1* gene located on chromosome 9q33.3 (1). Initially identified for its role in steroidogenesis (2), it is now known to be essential for gonadal and adrenal development by regulating key genes involved in steroid hormone production, gonadotropin expression, and Leydig cell function. In addition, it contributes to the development of the ventromedial hypothalamic nucleus (3). Variants in *NR5A1* lead to a wide range of clinical presentations, including differences in sex development, infertility, and adrenal insufficiency (4, 5, 6). Recently, its role in spleen development has been highlighted. Indeed, splenic abnormalities were reported in several patients carrying *NR5A1* variants, supporting the importance of assessing splenic function in clinical management (7, 8, 9).

The spleen is essential for immune regulation, connecting innate and adaptive immunity and protecting against infections. Hyposplenism refers to reduced spleen function, while asplenia is the complete absence of the spleen, usually after surgery. Both hyposplenism and asplenia increase the risk of severe infections, thromboembolism, and cancer (10). Due to high mortality, fulminating course, and resistance to common treatments for infections caused chiefly by encapsulated bacteria, vaccination and antibiotic prophylaxis are warranted in patients with asplenia or hyposplenism. Systematic and swift antibiotic therapy to targeting pneumococcus for any febrile episode is also recommended (11).

Various methods can be used to assess spleen function, but their sensitivity is often low (12). Quantification of pocked red blood cells (pRBC) is a simple, reproducible test that correlates with splenic scintigraphy (13, 14), making it the gold standard for detecting splenic dysfunction. However, its use in clinical practice remains limited by the need for specialised equipment (Nomarski optics). Fixing RBCs for analysis in expert centres could broaden its application (11).

The main aim of our study was to evaluate spleen function in French patients with *NR5A1* variants by measuring the prevalence and severity of hyposplenism using pRBC. We also aimed to describe spleen morphology and explore its relationship with functional impairment. Finally, we examined possible genotype–phenotype correlations related to splenic abnormalities.

## Materials and methods

### Patients and study design

This multicentre, cross-sectional study was nested within the prospective GR-Ex cohort (pathophysiological explorations of red blood cells; ClinicalTrials.gov identifier: NCT03541525), approved by the Ethics Committee (Comité de Protection des Personnes Ile de France II – Approval Number: 2016-A00114-47). Inclusion criteria for the GR-Ex cohort were as follows: to be affected or have a family history of disease related to red blood cells; for adults, to have signed an informed consent form; for minors or adults under legal protection, the form must be signed by both parents (for minors) or by the legal representative; and to be affiliated to health insurance. Exclusion criteria for the GR-Ex cohort were as follows: being deprived of liberty.

Patients were identified through a multicentre collaboration involving several national reference centres for disorders of sex development (DSD). All individuals carrying a pathogenic or likely pathogenic *NR5A1* variant, as well as their relatives with the same variant, with or without gonadal involvement, were invited to participate.

Ethically, this nested study was conducted within the national GR-Ex framework. All participants (or their legal representatives) provided written informed consent for inclusion in the GR-Ex cohort and for participation in the present study. Exclusion criteria for this nested study included the absence of an *NR5A1* variant and lack of informed consent. Participants were enrolled between September 2022 and July 2024.

Clinical and genetic data, pRBC counts, and radiological findings were retrieved from medical records. All data were anonymised. The severity of disorders of sex development (DSD) phenotypes was assessed using a modified external genitalia score (EGS) based on karyotype and external genitalia appearance at birth or before any genital surgery (15). Pituitary and adrenal function were also evaluated.

The primary outcome was the description (prevalence and severity) of morphological and functional hyposplenism in patients carrying pathogenetic/likely pathogenic *NR5A1* variants. The secondary outcome was to assess the relationship between spleen morphology (imaging) and functional impairment (pRBCs). Finally, exploratory analyses evaluated potential associations of hyposplenism with *NR5A1* genotype on the one hand, and with complete gonadal dysgenesis among patients with a 46,XY karyotype on the other.

### Genetic variant classification

Genetic *NR5A1* variants were described according to variant type, the change induced at the protein level, and its predicted functional impact on the protein. Variant impact was determined according to the Ensemble Variant Effect Predictor (16). Hence, high-impact variants were defined as frameshift, nonsense, or structural variants, while moderate-impact variants were defined as in-frame or missense variants. No low-impact variant (such as synonymous variant) of *NR5A1* was identified in our cohort. All patients carried ‘likely pathogenic’ or ‘pathogenic’ variants according to the 2015 ACMG classification. Variants were described following Human Genome Variation Society (HGVS) recommendations using the reference transcript (NM_004959.5) and protein (NP_004950.2) sequences.

### Morphological assessment

All patients underwent radiological assessment by ultrasound, CT scan, or MRI to explore the presence, size, and morphology of the spleen according to the clinical context.

### Pocked RBC quantification

Whole blood was collected in heparin-containing tubes, fixed in 0.1% PBS-buffered glutaraldehyde, and kept at 4°C until analysis. At least 300 RBCs were used to determine the proportion of pRBCs using the Leica microscope (DM1000 LED model) with oil-immersion objective (×1,000) and DIC (Nomarski optics), as described by Holroyde *et al.* (17). pRBCs under 7%, between 7 and 20%, or above 20% were indicative of normal, moderate hyposplenism, or absent splenic function (severe hyposplenism), respectively.

### Statistical analyses

Statistical analyses were performed using R 4.3.1 (https://www.r-project.org/). Univariate analyses were performed to describe the sample. Continuous variables were expressed as the median and interquartile range (IQR), categorical variables as the number, percentage, and 95% confidence intervals (95% CIs). Bivariate analyses were performed to compare groups. Qualitative variables were compared using *χ*^2^ tests and Fisher’s exact tests (FET), quantitative variables using Wilcoxon tests or Spearman’s correlation tests, and ordinal variables using Jonckheere–Terpstra tests. Genotype–phenotype analyses were primarily descriptive; any comparative tests are reported for information only in an exploratory context. Accordingly, no adjustments for multiple testing were applied to exploratory analyses, consistent with Bender & Lange (18). All tests were two-tailed. The significance threshold was set at *P* < 0.05.

## Results

### Clinical and paraclinical characteristics of the cohort

Thirty-four patients from twenty-one different families carrying heterozygous *NR5A1* variants were included in this study. Clinical and paraclinical characteristics of the cohort are summarised in Table 1. The median age was 18.5 years (IQR: 6–31 years). Nineteen (55.9%) patients exhibited a female phenotype. A total of 19 *NR5A1* genetic variants were identified: 10 substitutions in 20 patients, 5 insertion/deletion variants in 7 patients, and 3 structural variants in 5 patients. The precise genetic results for two patients (F7P1 and F9P1) were unavailable (Table 1). Twenty-four patients (70.6%) had a 46,XY karyotype. Among 46,XY patients, all had gonadal dysgenesis. Specifically, 9 (37.5% (95% IC: 21.2–57.3)) had 46,XY complete gonadal dysgenesis, 11 (45.8%) had a severe phenotype, and 4 (16.7%) had a mild phenotype according to EGS classification. All 46,XX patients had a female phenotype without gonadal dysgenesis according to EGS classification. Of note, four 46,XX patients showed premature ovarian insufficiency characterised by secondary amenorrhoea before the age of 40, associated with elevated gonadotropin levels confirmed by multiple assessments. None of these patients had adrenal or pituitary insufficiency.

**Table 1:** Clinical and genetic characteristics of patients. High-impact variants comprise frameshift, nonsense, and structural NR5A1 variants; moderate-impact variants comprise in-frame or missense NR5A1 variants. Spleen morphology was determined by ultrasound, CT scan, or MRI. CGD: complete gonadal dysgenesis; F: female; M: male; RBC: red blood cells; SV: structural variants. Patients’ ages are grouped into 5-year bands. y: years.

Patient ID	Age (y)	Sex	Karyotype	*NR5A1* genetic variant	Protein-level change	Variant type	Variant impact	DSD phenotype classification	Spleen morphology	Pocked RBC (%)
**Family 1**										
F1P1	(10–14)	F	46,XY	c.1084C>T	p.(Gln362Ter)	Nonsense	High	CGD	Polysplenia	34.5
**Family 2**										
F2P1	(30–34)	F	46,XX	c.680T>A	p.(Ile227Asn)	Missense	Moderate	Typical	Microsplenia	27.5
F2P2	(5–9)	F	46,XY					CGD	Polysplenia	22
F2P3	(0–4)	M	46,XY					Severe	Microsplenia	33
**Family 3**										
F3P1	(15–19)	M	46,XY	c.721C>T	p.(Arg241Trp)	Missense	Moderate	Mild	Normal	5.1
**Family 4**										
F4P1	(15–19)	F	46,XY	c.1271_1273del	p.(Val424del)	In-frame	Moderate	CGD	Normal	14.6
**Family 5**										
F5P1	(0–4)	M	46,XY	9q33.3 (127,176,244_127,319,364) × 1	p.?	SV	High	Severe	Normal	5.6
**Family 6**										
F6P1	(50–54)	F	46,XX	c.848G>T	p.(Cys283Phe)	Missense	Moderate	Typical	Asplenia	20
F6P2	(25–29)	F	46,XY					CGD	Normal	3.2
**Family 7**										
F7P1	(15–19)	M	46,XY	-	-	-	-	Severe	Normal	5.9
**Family 8**										
F8P1	(10–14)	M	46,XY	c.614dup	p.(Gln206ThrfsTer20)	Frameshift	High	Severe	Microsplenia	27.8
**Family 9**										
F9P1	(5–9)	M	46,XY	-	-	-	-	Severe	Normal	3
**Family 10**										
F10P1	(20–24)	F	46,XY	c.848G>T	p.(Cys283Phe)	Missense	Moderate	CGD	Microsplenia	28.2
**Family 11**										
F11P1	(20–24)	F	46,XY	c.1227C>A	p.(Tyr409Ter)	Nonsense	High	CGD	Polysplenia	53.2
F11P2	(55–59)	M	46,XY					Mild	Asplenia	52
**Family 12**										
F12P1	(0–4)	M	46,XY	Del (9) (q33.3q33.3)	p.?	SV	High	Severe	Normal	2.7
**Family 13**										
F13P1	(25–29)	F	46,XX	c.848G>T	p.(Cys283Phe)	Missense	Moderate	Typical	Normal	15
**Family 14**										
F14P1	(0–4)	M	46,XY	c.1342dup	p.(Arg448ProfsTer119)	Frameshift	High	Severe	Normal	17.7
**Family 15**										
F15P1	(0–4)	F	46,XY	c.175A>T	p.(Lys59Ter)	Nonsense	High	CGD	Normal	21.8
F15P2	(30–34)	F	46,XX					Typical	Normal	0.8
F15P3	(55–59)	F	46,XX					Typical	Normal	1.3
F15P4	(0–4)	F	46,XX					Typical	Normal	5
**Family 16**										
F16P1	(5–9)	M	46,XY	c.952G>A	p.(Gly318Ser)	Missense	Moderate	Mild	Normal	0
**Family 17**										
F17P1	(5–9)	F	46,XY	c.238_244 + 13del	p.(Leu80ProfsTer7)	Frameshift	High	CGD	Normal	4.5
**Family 18**										
F18P1	(50–54)	F	46,XX	c.983G>A	p.(Gly328Glu)	Missense	Moderate	Typical	Microsplenia	21.8
F18P2	(20–24)	M	46,XY					Severe	Normal	31.8
F18P3	(15–19)	M	46,XY					Severe	Normal	4.7
**Family 19**										
F19P1	(30–34)	F	46,XY	Deletion of exon 7	p.?	SV	High	CGD	Microsplenia	22
F19P2	(35–39)	F	46,XX					Typical	Normal	6.2
F19P3	(30–34)	F	46,XX					Typical	Microsplenia	15.6
**Family 20**										
F20P1	(0–4)	M	46,XY	c.630_640del	p.(Tyr211ArgfsTer11)	Frameshift	High	Severe	Microsplenia	69.6
F20P2	(35–39)	F	46,XX					Typical	Asplenia	62.2
F20P3	(30–34)	M	46,XY					Mild	Asplenia	63.4
**Family 21**										
F21P1	(5–9)	M	46,XY	c.1015C>T	p.(Gln339Ter)	Nonsense	High	Severe	Normal	16

All participants had spleen imaging performed. Nineteen patients (55.9% (95% IC: 39.5–71.1)) had normal spleen morphology, while 15 (44.1% (95% IC: 28.9–60.5)) showed abnormal findings. More specifically, 4 (11.8% (95% IC: 4.7–26.7)) exhibited asplenia (absence or complete atrophy of the spleen), while the remaining 11 (32.3% (95% IC: 19.1–49.2)) had microsplenia or polysplenia. pRBCs were quantified in all patients. Twenty-one (61.8% (95% IC: 45.0–76.1)) had hyposplenism, while 13 (38.2% (95% IC: 23.9–55.0)) had normal splenic function. More precisely, 16 (47.1% (95% IC: 31.5–63.3)) showed severe hyposplenism (i.e. pRBCs >20%), while five (14.7% (95% IC: 6.4–30.1)) had moderate hyposplenism (Table 1). There was no correlation between patient age and the presence of hyposplenism (*r* = 0.11, *P* = 0.54).

Among the 34 patients, three developed severe meningitis in adulthood, before the genetic diagnosis of the NR5A1 mutation, which is responsible for hyposplenism. One patient developed purpura fulminans, resulting in quadruple limb amputation, while the other two sustained severe neurological sequelae following the infection.

### Association between splenic morphological characteristics and genetic characteristics

There was no significant difference in the number of patients with morphological spleen anomalies between 46,XY (41.7% (95% CI: 24.5–61.2)) and 46,XX karyotypes (50.0% (95% CI: 23.7–76.3)) (*χ*^2^ = 0.20, df = 1, *P* = 0.66). Among 46,XY-karyotype patients, there was no significant difference in the proportion of splenic morphological anomalies between those presenting with male (33.3% (95% CI: 15.2–58.3)) versus female phenotypes (55.5% (95% CI: 26.7–81.1)) (FET, *P* = 0.40).

No association between the predicted functional impact of *NR5A1* genetic variants and the presence of morphological spleen anomalies was observed (Table 2). Patients carrying the p.(Ile227Asn), p.(Gln362Ter), p.(Tyr409Ter), p.(Gln206ThrfsTer20), or p.(Tyr211ArgfsTer11) *NR5A1* variants presented with morphological spleen anomalies (Table 1).

**Table 2:** Relationship between morphological spleen abnormalities, splenic function markers, and the predicted impact of genetic variants in *NR5A1* patients. High-impact variants comprise frameshift, nonsense, and structural *NR5A1* variants; moderate-impact variants comprise in-frame or missense *NR5A1* variants. Splenic dysmorphia was defined as microsplenia, polysplenia, atrophic spleen, and asplenia. pRBC: pocked red blood cell. Abnormal spleen function was defined by a pRBC >7%. *n*: number of patients. *χ*^2^ tests were performed.

	Variant impact		*P*
	High (*n* = 19)	Moderate (*n* = 13)	
**Spleen morphology**			
Normal spleen	10/19 (52.6%)	7/13 (53.8%)	0.95
Spleen dysmorphic or absent	9/19 (47.4%)	6/13 (46.2%)	
**Pocked RBC levels**			
Elevated levels of pRBC	12/19 (63.2%)	9/13 (69.2%)	0.72

### Association between spleen function and genetic characteristics

There was no significant difference in the number of patients with splenic functional disturbance between 46,XY (62.5% (95% CI: 42.7–78.8)) and 46,XX karyotypes (60.0% (95% CI: 31.3–83.2)) (FET, *P* = 1.00). Among 46,XY-karyotype patients, there was no significant difference in the proportion of splenic functional disturbance between those presenting with male (53.3% (95% CI: 30.1–75.2)) versus female phenotypes (77.8% (95% CI: 45.3–93.7)) (FET, *P* = 0.39).

No association was observed between the predicted functional impact of *NR5A1* genetic variants and the presence of immunological anomalies (Table 2). Patients carrying the p.(Ile227Asn), p.(Gln362Ter), p.(Tyr409Ter), p.(Gln339Ter), p.(Val424del), p.(Gln206ThrfsTer20), p.(Arg448ProfsTer119), or p.(Tyr211ArgfsTer11) *NR5A1* variants presented with immunological anomalies (Table 1).

### Association between spleen function and spleen morphology

All patients with abnormal splenic imaging had abnormal pRBCs, whereas six patients with normal imaging also had abnormal pRBCs. There were thus more cellular abnormalities (pRBC >7%) in patients with morphological spleen anomalies compared to patients with normal spleen (Table 3). Accordingly, patients with morphological spleen anomalies had higher pRBC levels (median: 28.2; IQR: 22.0–52.6) compared to patients with normal spleen imaging (5.1 (3.1–14.8); Wilcoxon test, *P* < 0.001).

**Table 3:** Relationship between elevated pRBC levels and morphological spleen abnormalities in *NR5A1* patients. Spleen morphology was determined by ultrasound, CT scan, or MRI. Splenic dysmorphia was defined as microsplenia, polysplenia, atrophic spleen, and asplenia. Abnormal spleen function was defined by a pRBC >7%. pRBC: pocked red blood cell. *n*: number of patients. A *χ*^2^ test was performed. Bold indicates strong statistical significance.

Spleen morphology	Elevated levels of pocked RBC	*P*
Normal (*n* = 19)	6/19 (31.6%)	**4.6 × 10^−^** ^**5**^
Spleen abnormalities (*n* = 15)	15/15 (100%)	

**Figure 1:**
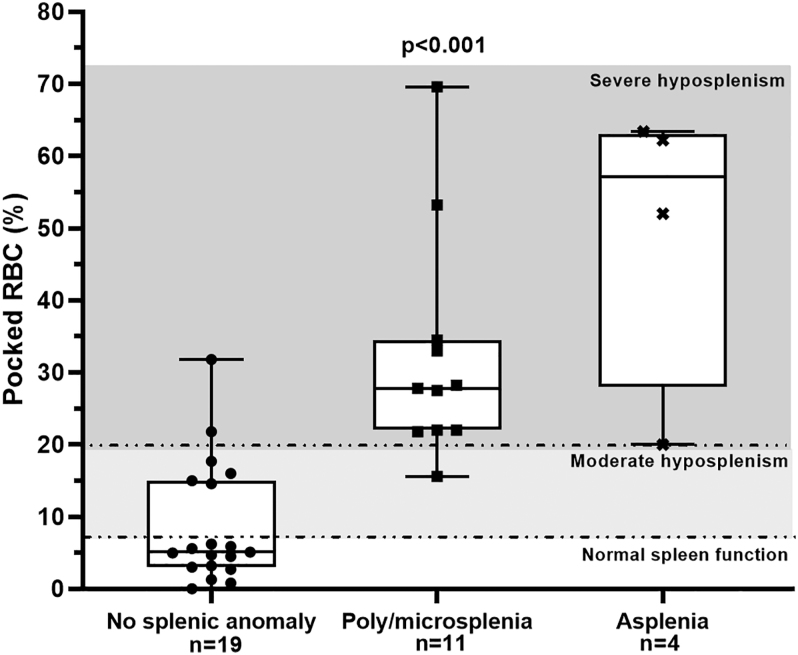
Pocked RBCs according to spleen morphology in *NR5A1* patients. Asplenia was defined by an absent or atrophic spleen – normal spleen function (white zone) was defined by a pRBC <7%, moderate hyposplenism (light grey zone) by a pRBC between 7 and 20%, and severe hyposplenism (dark grey zone) by a pRBC >20%. Spleen morphology was determined by ultrasound, CT scan, or MRI. *n*: number of patients; RBC: red blood cell. A Jonckheere-Terpstra test was performed (the Jonckheere–Terpstra test assessed an ordered trend across spleen-morphology categories with the *a priori* order ‘normal < micro/polysplenia < asplenia’).

A qualitative association was observed between spleen function and the presence of functional abnormalities (Fig. 1). Indeed, among patients with normal spleen morphology, 13/19 (68.4% (95% CI: 46.0–84.6)) had normal spleen function, 4/19 (21.1% (95% CI: 8.5–46.3)) had moderate hyposplenism, and 2/19 (10.5% (95% CI: 2.9–31.4)) had severe hyposplenism. Among those with micro/polysplenia, 1/11 (9.1% (95% CI: 1.6–37.8)) had moderate hyposplenism and 10/11 (90.9% (95% CI: 62.3–98.4)) had severe hyposplenism. Among those with asplenia, 4/4 (100%) had severe hyposplenism (FET; 3.1 × 10^−6^). Moreover, a quantitative relationship was observed between pRBC levels and the severity of morphological spleen abnormalities (*P* < 0.001) (Fig. 1). Patients with normal spleen morphology exhibited significantly lower pRBC levels compared to those with micro- or polysplenia, who, in turn, had lower pRBC levels than patients with asplenia. However, 6 of 19 patients with normal spleen morphology had abnormal pRBC levels, indicating some degree of hyposplenism.

## Discussion

Our study demonstrates a high prevalence of splenic dysfunction among individuals carrying *NR5A1* variants in our cohort. Functional hyposplenism affected a substantial proportion of participants, with nearly half exhibiting a severe form. Morphological abnormalities were also frequently observed, including several cases of complete asplenia. Our findings are consistent with those of the European cohort published in 2024 by Cools *et al.*, who assessed splenic function in 22 individuals with *NR5A1* variants (9). Their results showed that 50% of participants, with or without a difference in sex development, had anatomical abnormalities of the spleen, and that the number of non–class-switched memory B cells – a spleen-specific subset of B lymphocytes – was significantly reduced compared to healthy controls (9). Although the methods used to assess splenic function differed between the two cohorts, the overall findings are highly concordant.

In our cohort, all patients with structural splenic anomalies also exhibited functional impairment. However, a considerable number of individuals with hyposplenism had normal imaging findings, highlighting that radiological evaluation alone may fail to detect functional splenic dysfunction.

The absence of a genotype–phenotype correlation with splenic abnormalities is consistent with prior studies showing the variable expressivity of *NR5A1* variants, particularly in gonadal and adrenal phenotypes (5, 6, 19, 20). This supports the hypothesis of multifactorial pathogenicity, possibly involving modifier genes or oligogenic interactions (4, 21, 22, 23, 24), as well as epigenetic and environmental factors (6). In addition to these oligogenic and environmental influences, dosage sensitivity may also contribute to the phenotypic variability. The seminal study that first established the role of SF-1 in splenic development reported a homozygous loss-of-function variant, whereas virtually all subsequent cases have involved heterozygous variants. Notably, SF-1 displays a dose-dependent effect on the transcription of its target genes in animal models (25), supporting a gene-dosage–sensitive mechanism. Thus, depending on the functional severity of a given variant, a heterozygous alteration may already be sufficient to produce hyposplenism, while homozygosity would be expected to result in a more severe or fully penetrant phenotype. Accordingly, in the previously largest cohort describing splenic dysfunction in *NR5A1* patients before ours, almost all individuals carried heterozygous variants and appeared to have a less severe splenic phenotype compared with the homozygous patient (9). Nevertheless, as our cohort included only heterozygous variants, this mechanism remains inferred rather than directly demonstrated and should be interpreted with caution.

*NR5A1*’s role in spleen development is increasingly supported by molecular and clinical evidence (7, 8, 26). Classically, SF-1 regulates the transcription of TLX1, a homeobox gene essential for splenic organogenesis, and reduced TLX-1 expression has been associated with specific *NR5A1* variants located in the N-terminal DNA-binding domain (7, 27). In addition, C-terminal *NR5A1* variants have been reported to cause transcriptional dysregulation through alterations of the ligand-binding domain (28, 29). These mechanisms may contribute to the structural splenic abnormalities observed in affected individuals. However, in our cohort, we found no correlation between the type or location of *NR5A1* variants and the clinical phenotype, consistent with previous findings in larger series (6).

Clinically, hyposplenism carries a high risk of invasive infections, particularly with encapsulated organisms such as *Streptococcus pneumoniae*, *Haemophilus influenzae*, and *Neisseria meningitidis*. In our cohort, three adults had severe neurological or motor sequelae due to invasive infections before diagnosis and without prophylactic care, underlining the importance of early identification and preventive management. All patients diagnosed with hyposplenism in this study were informed of their immunological risk, received appropriate vaccinations, and were educated about managing potential infectious or thromboembolic symptoms.

We used both morphological and functional assessments, abdominal imaging and pRBC quantification, which are complementary. The pRBC test, although requiring specific equipment, is reproducible and sensitive, particularly in detecting mild or subclinical dysfunction (11, 14). In patients with normal imaging, pRBC analysis remains critical. Additional markers such as CD27+ IgM memory B cells (26) may provide further evaluation when pRBC levels are borderline or inconclusive.

Considering the frequency of radiological abnormalities, our study may help refine screening and management strategies for these patients.

In our cohort, three adult patients suffered from severe neurological and/or motor sequelae caused by invasive infections that occurred before diagnosis with splenic impairment and therefore in the absence of any preventive measures. This highlights the importance of systematic screening for hyposplenism in patients carrying *NR5A1* variants, as well as the application of preventive measures when necessary.

No correlation was observed between the presence or severity of gonadal anomalies and splenic dysfunction. This suggests that *NR5A1* variants may confer risk of hyposplenism independently of gonadal phenotype, reinforcing the need for splenic evaluation in all *NR5A1* carriers, including asymptomatic individuals. This decoupling likely reflects tissue-restricted regulatory logic and developmental timing. SF-1 regulates targets in the gonad (e.g. SOX9, steroidogenic genes) via sets of co-regulators and regulatory elements that differ from those engaged for TLX1 or CYP26B1 during splenic development. Consequently, the same variant may disproportionately disrupt one network compared with the other. Moreover, the spleen has a narrow embryonic window of dependence on TLX1 (and downstream retinoic acid signalling), whereas the gonad benefits from redundant/compensatory pathways (e.g. SRY/SOX9, WT1/GATA4 networks), which can mask the effect of the same variant (25). Of note, other conditions related to the *NR5A1* variant were absent in our cohort, including adrenal insufficiency, which remains rare despite its early description in *NR5A1* patients (30). Indeed, only five cases of adrenal insufficiency have been observed in the largest SF-1/*NR5A1* cohort of 197 patients (6).

A major strength of this study lies in the standardised evaluation of both spleen morphology and function, performed in a single reference laboratory, which enhances consistency and comparability across patients. In addition, pRBC counts were measured using standardised procedures, ensuring uniformity in the assessment of haematologic parameters. The combined evaluation of spleen morphology and function provides a more comprehensive picture of splenic involvement, which is particularly relevant in the context of *NR5A1* variants. The age distribution was also relatively balanced, which helps reduce age-related confounding.

However, several limitations should be acknowledged. The retrospective collection of some data, along with the multicentric design, may have introduced heterogeneity in data collection and limited the accuracy of certain clinical variables. The sample size remains relatively small, reflecting the rarity of *NR5A1* variants, which reduces statistical power, particularly for genotype–phenotype correlation analyses. Potential selection bias may also be present, especially given the inclusion of several familial cases, which may overrepresent certain phenotypic features. These factors may limit the generalisability of the findings and call for future prospective studies with larger cohorts.

## Conclusion

In conclusion, our study contributes to the current understanding of splenic function in individuals carrying an *NR5A1* variant. More than half of the patients in our cohort with a pathogenic or likely pathogenic *NR5A1* variant present with hyposplenism, regardless of genotype or gonadal phenotype. Therefore, assessing splenic function is essential for the follow-up of these patients. Our data suggest that proactive clinical management and careful monitoring could help reduce the risks associated with hyposplenism. Future studies, including larger cohorts, ideally through international collaborations, are needed to validate these findings and better estimate the prevalence and severity of hyposplenism in this population. In addition, mechanistic studies exploring the link between NR5A1 gene variants and splenic function could provide new insights into the underlying pathophysiology.

## Declaration of interest

Khadidja Fouatih, Camille Roussel, Maryse Cartigny, Muriel Houang, Lise Duranteau, Zeina Chakhtoura, Anne-Sophie Lambert, Marie-Agathe Trouvin, Barbara Girerd, Ekaterina Belozertseva, Delphine Borgel, Stephanie Franchi-Abella, Jérôme Bouligand, Maureen Lopez, Kenneth Chappell, Pierre Buffet, Ines Belguith, Claire Bouvattier and Abd El Kader Ait Tayeb have no conflict of interest to disclose.

## Funding

This work did not receive any specific grant from any funding agency in the public, commercial, or not-for-profit sector.

## Author contribution statement

KF and CR conceptualised the study. KF collected clinical data and wrote the manuscript. CR performed biological analyses and contributed to manuscript revision. SF-A was responsible for radiological image analysis. CB and AEKAT supervised the project and provided critical revision of the manuscript. AEKAT also contributed to data analysis and study design. MCa, MH, LD, ZC, A-SL, M-AT, BG, EB, DB, JB, ML, KC, PB, and IB contributed to patient inclusion and critical review of the manuscript. All authors reviewed and approved the final version of the manuscript.

## Data availability

The datasets generated and analysed in the current study are not publicly available due to the inclusion of sensitive data and to ensure patient anonymity. They are available from the corresponding author upon reasonable request.
